# Different Aspects of Pneumatosis Intestinalis

**DOI:** 10.7759/cureus.97041

**Published:** 2025-11-17

**Authors:** Helen Bolanaki, Ioannis Tzimagiorgis, Athanasia Mitsala, Anastasios J Karayiannakis

**Affiliations:** 1 Second Department of Surgery, University Hospital of Alexandroupolis, Alexandroupolis, GRC; 2 Second Department of Surgery, Democritus University of Thrace, Medical School, Alexandroupolis, GRC

**Keywords:** chemotherapy, complications, intestinal obstruction, intestinal perforation, pneumatosis intestinalis, small bowel pneumatosis

## Abstract

Pneumatosis intestinalis (PI) is a rare condition characterized by gas-filled cysts within the wall of the gastrointestinal tract with variable presentation. We present two patients with pneumatosis intestinalis different in their clinical presentation, clinical course, and treatment. A 79-year-old male with a history of chronic obstructive pulmonary disease presented with an acute abdomen. Clinical findings and abdominal radiography suggested perforation of the digestive tract. During emergency laparotomy, multiple cystic bubbles and perforation were found in the terminal ileum, and the affected part of the ileum was resected. The second patient was a 64-year-old female under adjuvant chemotherapy with cisplatin and paclitaxel for ovarian adenocarcinoma. She presented with signs of bowel obstruction. Abdominal computed tomography was diagnostic for intestinal pneumatosis. She was successfully treated conservatively. These two cases highlight the varied clinical presentation of pneumatosis intestinalis and differences in its treatment. High clinical suspicion, cautious judgment of clinical and laboratory findings, and careful assessment of the imaging findings are necessary for accurate diagnosis and appropriate management.

## Introduction

Pneumatosis intestinalis (PI) is a rare condition characterized by the presence of gas-filled cysts in the subserosa and/or submucosa of the small and large bowel [1]. PI is increasingly detected with the wide use of computed tomography; however, the etiology and pathogenesis are poorly understood. The condition can be primary or secondary related to underlying pathological conditions such as pulmonary and autoimmune diseases, chemotherapy, and organ transplantation, or digestive disorders associated with abnormal mucosal permeability like inflammatory bowel disease, and bowel obstruction or ischemia [1]. Manifestations of PI are very heterogeneous, varying from asymptomatic, incidentally detected cases to potentially life-threatening conditions like bowel ischemia, necrosis, or perforation [1, 2]. The wide range of clinical manifestations and etiologies poses diagnostic and treatment challenges to clinicians.

Here, we present two cases of pneumatosis intestinalis, different in their onset, clinical presentation, and clinical course, and different treatment approaches, which highlight these challenges.

## Case presentation

**Figure 1:**
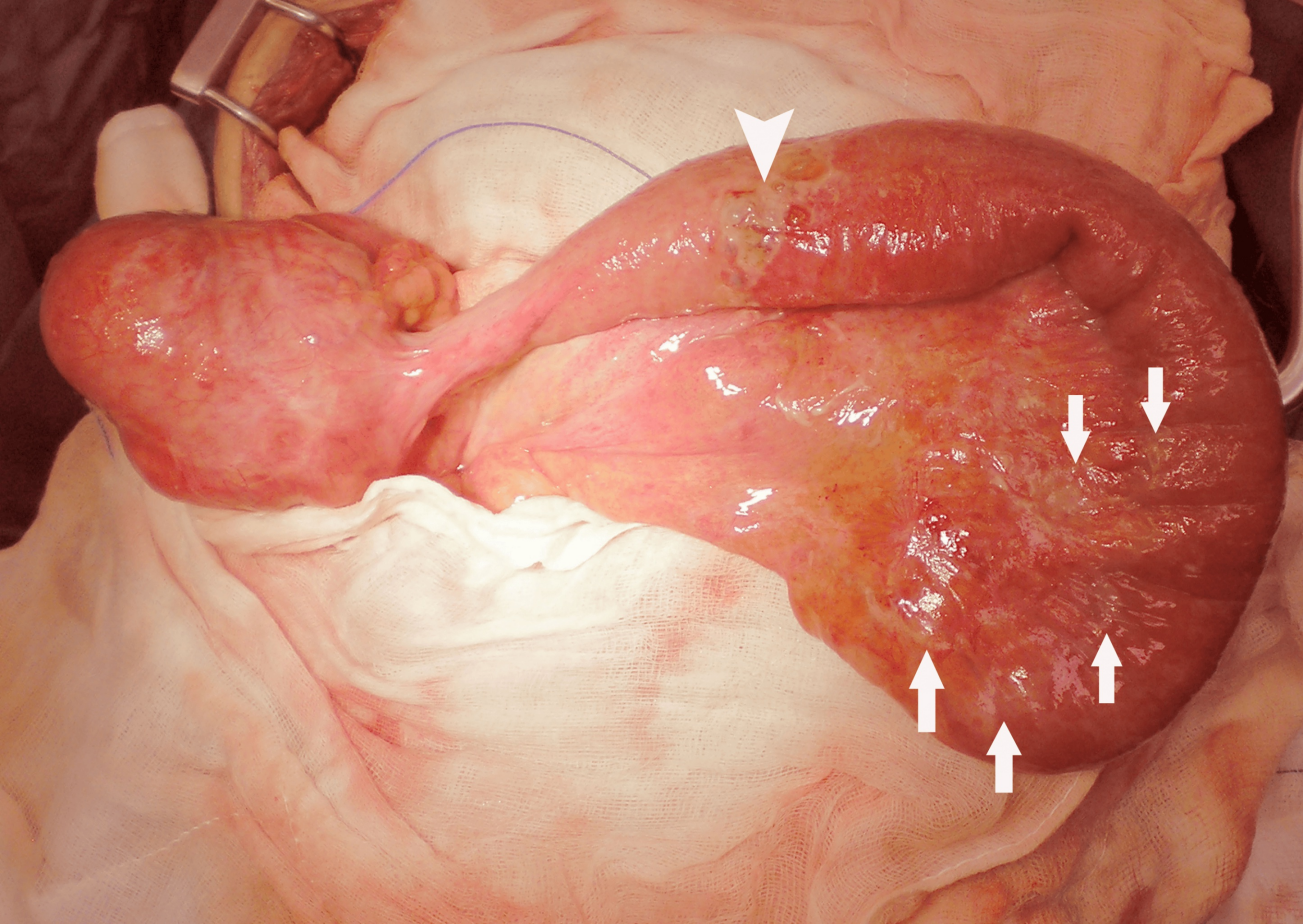
Intraoperative photograph. Showing thickened wall of the terminal ileum with cysts on its surface and on the mesentery (arrows). The cyst with ischemia and perforation is shown (arrowhead).

The first patient was a 79-year-old male who presented to the emergency department with severe, acute abdominal pain of sudden onset. He had a history of chronic obstructive pulmonary disease and was under treatment with a combination of inhaled corticosteroids and long‐acting bronchodilators (fluticasone and salmeterol) for many years. The patient was afebrile with normal vital signs. Abdominal examination revealed diffuse tenderness, abdominal wall guarding, rebound tenderness, and diminished bowel sounds. A laboratory analysis revealed elevated white blood cell counts at 12,000/microliter and elevated C-reactive protein levels at 2.3 mg/dL, but was otherwise unremarkable. An abdominal radiography showed free gas under the right dome of the diaphragm. Based on these findings, perforation of the digestive tract was considered, and he underwent an emergency laparotomy. Exploration of the abdominal cavity revealed a small amount of dark-colored fluid, and samples were taken for cultures. An ischemic lesion with perforation of the terminal ileum was found about 15 cm from the ileocecum. Multiple cystic bubbles were present on the adjacent intestinal wall at a length of 20 cm and also on the mesentery (Figure 1).

The diagnosis of pneumatosis cystoides intestinalis was made. The affected part of the ileum was resected, and an end-to-end anastomosis was performed. According to the histopathology report, there were multiple subserosal cysts surrounded by neutrophils, lymphocytes, and multinucleated giant cells. There was also diffuse edema in the submucosa and muscularis, while the mucosa was normal. Cultures samples failed to grow any pathogens. The postoperative course of the patient was smooth, and he was discharged from the hospital six days later.

**Figure 2:**
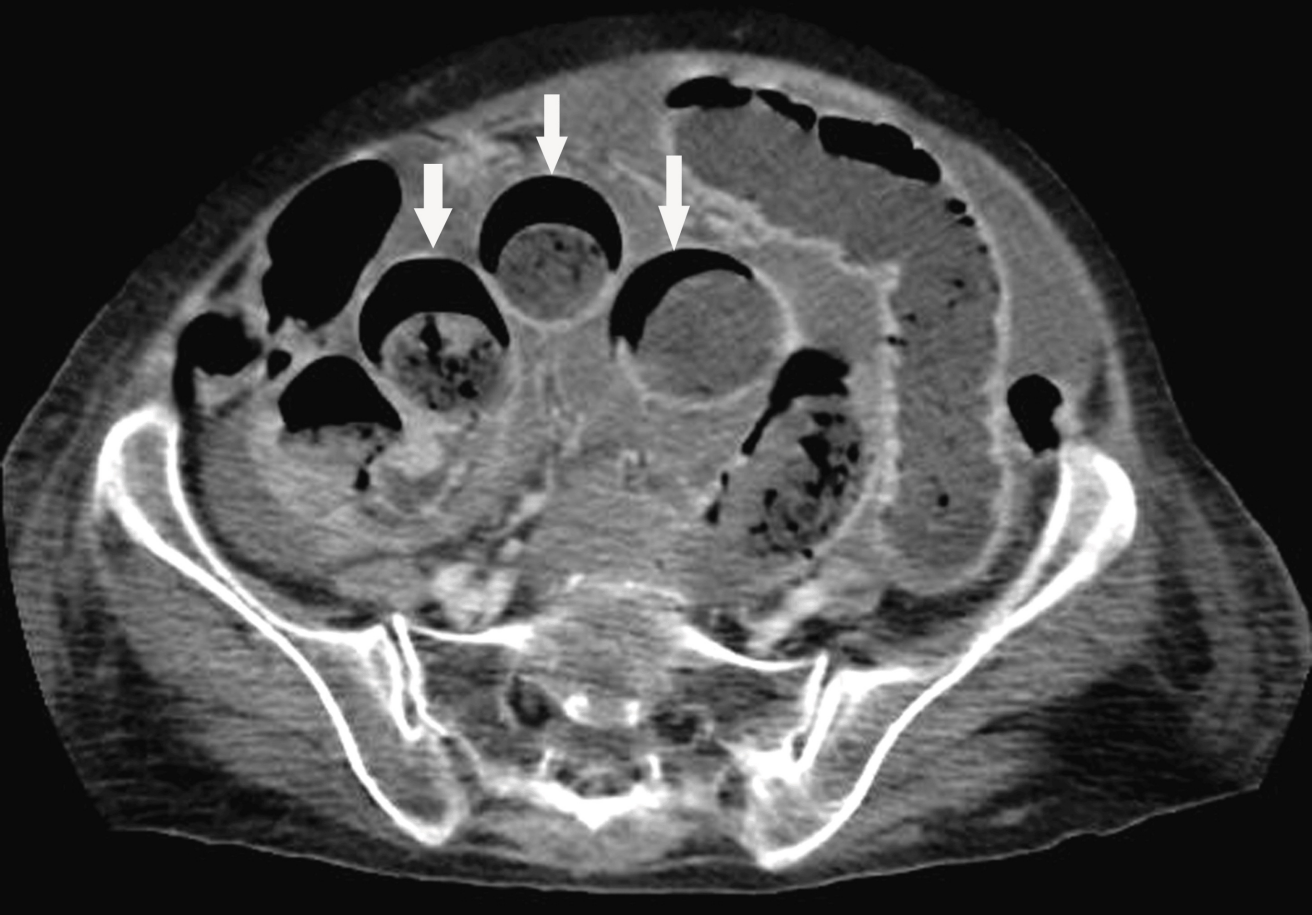
Axial contrast-enhanced CT scan of the abdomen. Showing significant curved-shaped accumulation of gas in the wall of the jejunum (arrows).

The second patient was a 64-year-old female with a history of total abdominal hysterectomy and bilateral salpingo-oophorectomy for ovarian adenocarcinoma who was on adjuvant chemotherapy with cisplatin and paclitaxel. One week after her third cycle of chemotherapy, she was admitted with breathing difficulty, excessive bilious vomiting, and inability to pass gas or flatus over the last two days. Physical examination revealed abdominal distension with diffuse mild tenderness, decreased bowel sounds, and signs of ascites. There were no signs of peritonitis, and she was afebrile. Laboratory findings showed decreased hemoglobin (8.7 g/dL) and white blood cell counts (3.7x109/L), and decreased protein (4.6 g/dL) and albumin (2.3 g/dL) levels, apparently attributed to the chemotherapy. An abdominal radiography showed increased air within the bowel but not air-fluid levels. Based on symptoms, physical and radiographic findings, bowel obstruction was suggested. However, contrast-enhanced computed tomography of the abdomen showed the presence of ascites and curvilinear accumulation of air within the small bowel wall (Figure 2). There were no findings of portal air, bowel necrosis, or perforatio,n and the diagnosis of intestinal pneumatosis was made.

The patient was treated conservatively by discontinuing oral feeding and nasogastric tube insertion. Crystalloid and albumin solutions, along with furosemide, were administered. Prophylactic piperacillin/tazobactam and low molecular weight heparin were also administered. The patient remained afebrile without any signs of peritonitis. Over the next three days, her symptoms gradually resolved, bowel motility was restored, and oral feeding was commenced.

## Discussion

Pneumatosis intestinalis is a rare condition characterized by the presence of cysts filled with gas in the subserosa and/or submucosa of the small and large bowel. Pneumatosis intestinalis occurs in approximately 0.03% of the population, with prevalence among older populations [1]. The condition is usually benign and often found incidentally on imaging and is increasingly detected with the wide use of computed tomography. Approximately 15% of PI cases are primary, and 85% are secondary due to an underlying pathology. PI can develop in any portion of the gastrointestinal tract, but is most commonly found in the small bowel (36.4%), followed by colon and rectum (29.7%) [1].

The etiology and the pathogenesis of PI are largely unknown, with multiple factors implicated; however, disruption of the mucosal integrity plays a pivotal role in all cases. Several theories have been proposed to explain the accumulation of gas into the bowel wall. The pulmonary theory is based on the observed association between PI and pulmonary diseases such as asthma, emphysema, and chronic obstructive pulmonary disease. It suggests that alveolar rupture results in gas migration through the mediastinal vessels caudally into the retroperitoneum and finally to the mesentery of the bowel. It can also occur because of increased intra-abdominal pressure associated with chronic cough or pulmonary obstruction. Poor oxygen delivery in the intestinal mucosa in patients with low arterial oxygen tension because of pulmonary disease may also compromise the mucosal barrier. In our first case, the patient was suffering from chronic obstructive pulmonary disease and his treatment included inhaled corticosteroids for a long time. Steroids may cause lymphoid depletion and mucosal barrier disruption, resulting in propagation of intraluminal gas through the intestinal wall. Similar reports have been published previously [3-6].

The mechanical theory supports the invasion of intraluminal gas into the bowel wall as a result of increased intraluminal pressure, mucosal damage, or a combination of both. Disruption of the mucosal and immune barriers from mechanical causes, inflammatory bowel disease, immunosuppressive and cytotoxic therapy may promote intramural gas diffusion. Our second patient was under adjuvant treatment with cisplatin and paclitaxel for an operated ovarian adenocarcinoma. Several chemotherapeutic agents, such as 5-Fluorouracil, doxorubicin, etoposide, docetaxel, irinotecan, and cisplatin, have been reported to associate with PI [7-9]. Cancer patients are prone to immunosuppression. The intestinal mucosa is characterized by a high cellular turnover and is very sensitive to chemotherapeutics. It is conceivable that mucosal barrier damage may occur during chemotherapy, thus permitting the invasion of intraluminal gas into the bowel wall.

The third, bacterial theory, is supported by reports of PI resolution after administration of antibiotics. It supports overgrowth of bacteria such as Clostridia and Escherichia species and direct invasion of the intestinal wall or overproduction of gas that migrates into the intestinal wall through a compromised mucosal barrier as a possible mechanism for PI [6].

The clinical presentation of PI is heterogeneous, varying from incidentally detected cases to potentially life-threatening complications [10]. The most common symptoms are diarrhea, bloody stools, abdominal pain, constipation, loss of appetite, weight loss, and tenesmus. These are nonspecific symptoms depending on whether PI is primary or secondary and may easily mislead the correct diagnosis. Primary PI usually results in mild abdominal symptoms, whereas secondary PI may present with life-threatening conditions like bowel ischemia, necrosis, perforation, and pneumoperitoneum, occurring in approximately 3% of cases [1, 2].

Careful history evaluation for previous surgical or endoscopic procedures, gastrointestinal, pulmonary, autoimmune, inflammatory, and malignant diseases, and diabetes, as well as steroid therapy, immunotherapy, or chemotherapy, may reveal an underlying cause of PI in up to 52% of cases and help with accurate diagnosis [1].

Abdominal radiographs, ultrasonography, computed tomography, magnetic resonance imaging, and endoscopy have been used to diagnose pneumatosis intestinalis [11, 12]. Plain radiographs may reveal linear, curvilinear, or cystic radiolucencies within the intestinal wall or free intraperitoneal air in approximately two-thirds of cases. Ultrasonography is more commonly applied to the pediatric population and may show linear or ring form echogenic areas in the intestinal wall, and may also detect air in the portal vein. Magnetic resonance has been rarely used for the diagnosis of PI. Colonoscopy may reveal bubble-like cystic lesions, which need to be distinguished from other lesions such as polyps, tumors, or Crohn’s disease. Computed tomography is the gold standard imaging, having a high sensitivity in diagnosing PI. Typically, it reveals low-density cystic, linear, or bubble-like forms of intramural gas. In addition, it allows the detection of additional associated pathologies and helps to distinguish complicated from uncomplicated cases of PI [2, 11, 12].

Intestinal pneumatosis may result in life-threatening complications such as pneumoperitoneum and peritonitis due to bowel ischemia, necrosis, and perforation occurring in approximately 3% of cases with a mortality rate of 50% to 75% [1]. Distinguishing these cases is important for appropriate treatment [13-15]. Clinical presentation, thorough evaluation of CT findings, careful clinical examination, and findings from laboratory tests are important for distinguishing complicated PI. Fever, obstructive symptoms, signs of peritonitis, sepsis and shock, older age, evidence of portal venous gas on CT, leukocytosis, elevation of C-reactive protein, lactic acidosis, are worrisome features and poor prognostic factors necessitating surgical management [1, 2]. In the absence of such findings, PI is considered non-complicated, and up to 50% of patients are usually treated conservatively, as in our second patient.

Conservative measures include cessation of any medications known to associate with PI or with increased formation of intestinal gas, gastrointestinal “rest’, fluid and electrolyte supplementation, parenteral nutrition, or elemental diet. Antibiotics, most commonly metronidazole, are given to inhibit the growth of intestinal pathogens and therefore reduction of gas production. Ornidazole and Bifidobacterium have also been used to modify the intestinal flora. Inhalational or hyperbaric oxygen therapy has been used to increase the pressure gradient of the cystic gas and therefore facilitate gas diffusion outside the intestinal wall. However, optimal oxygen concentrations, route of administration, duration of treatment, and effectiveness of oxygen therapy have not been resolved [16]. Conservative measures should continue until clinical and imaging resolution of PI. Close observation and careful patient monitoring are necessary for the early detection of emerging symptoms and signs suggestive of complications, and repeated imaging is recommended for high-risk patients and in case of clinical worsening.

Surgical treatment is considered for complicated cases and is necessary for 36.9% of PI patients [1]. Clinical signs of sepsis or peritonitis, older age, leukocytosis, lactic acidosis, free air or fluid in the abdomen, and the presence of portal venous gas are indications for surgical intervention. Definitive surgery should be performed when intestinal ischemia, necrosis, or perforation is found during laparotomy [17]. This was the case in our first patient, where clinical presentation, findings of peritonitis from clinical examination, and findings from laboratory tests, along with the radiographic evidence of free air under the right hemidiaphragm, suggested bowel perforation, and he underwent an emergency exploratory laparotomy. Ischemia and rupture of a cystic bubble were found, and the diseased intestinal segment was resected.

However, it should be emphasized that PI can be associated with the presence of free intraperitoneal air without bowel perforation. This condition, also known as “non-surgical” or “benign” pneumoperitoneum, is not associated with bowel perforation. Rupture of a cyst is the source of free intraperitoneal air without actual bowel perforation. This condition can be managed conservatively and should be distinguished from the life-threatening true bowel perforation to avoid misdiagnosis and unnecessary surgery [13-15]. This is a challenging task and not always easy. High clinical suspicion, cautious judgment of clinical and laboratory findings, along with the imaging findings, may help with proper management.

## Conclusions

Pneumatosis intestinalis either primary or secondary is a rare condition presenting with a wide variety of manifestations. Proper imaging along with cautious clinical judgment is necessary for accurate diagnosis and appropriate management.
